# The effects of sexual violence experienced in childhood and adolescence on undergraduate students

**DOI:** 10.11606/s1518-8787.2020054002576

**Published:** 2020-11-27

**Authors:** Flávia Calanca da Silva, Aline Monge, Carlos Alberto Landi, Gabriel Amaral Zenardi, Denise Chrysostomo Suzuki, Maria Sylvia de Souza Vitalle

**Affiliations:** I Universidade Federal de São Paulo Escola Paulista de Medicina Departamento de Pediatria São PauloSP Brasil Universidade Federal de São Paulo. Escola Paulista de Medicina. Departamento de Pediatria. São Paulo, SP, Brasil; II Universidade Federal de São Paulo Escola Paulista de Medicina Programa de Pós-Graduação em Saúde Coletiva São PauloSP Brasil Universidade Federal de São Paulo. Escola Paulista de Medicina. Programa de Pós-Graduação em Saúde Coletiva. São Paulo, SP, Brasil; III Universidade Federal de São Paulo Escola de Filosofia, Letras e Ciências Humanas Programa de Pós-Graduação em Educação e Saúde GuarulhosSP Brasil Universidade Federal de São Paulo. Escola de Filosofia, Letras e Ciências Humanas. Programa de Pós-Graduação em Educação e Saúde. Guarulhos, SP, Brasil; IV Faculdade de Ciências Médicas da Santa Casa de São Paulo Departamento de Atenção Primária São PauloSP Brasil Faculdade de Ciências Médicas da Santa Casa de São Paulo. Departamento de Atenção Primária. São Paulo, SP, Brasil; V Universidade Nove de Julho Faculdade de Medicina Módulo de Pediatria São PauloSP Brasil Universidade Nove de Julho. Faculdade de Medicina. Módulo de Pediatria. São Paulo, SP, Brasil; VI Universidade Federal de São Paulo Escola Paulista de Medicina São PauloSP Brasil Universidade Federal de São Paulo. Escola Paulista de Medicina. São Paulo, SP, Brasil

**Keywords:** Adolescent, Young Adult, Sexual Behavior, Child Abuse, Sexual, Sex Offenses, Mental Disorders, epidemiology, Substance-Related Disorders

## Abstract

**OBJECTIVE:**

This study aims to investigate the prevalence of adolescents and young adults who were victims of sexual violence at some point in their lives and to compare the presence of depressive and anxious symptoms, quality of life, and use of alcohol, tobacco, and illegal drugs among this population and those who were not abused.

**METHODS:**

Validated questionnaires and instruments were applied in a group of university students to assess: sexual profile and behavior, socioeconomic status, presence or not of sexual violence (Questionnaire on Exposure to Traumatizing Events), depressive (Beck Depression Inventory) and anxious symptoms (Beck Anxiety Inventory), quality of life (World Health Organization's Quality of Life Assessment) and the use or abuse of tobacco, alcohol, and illegal drugs (Smoking, Alcohol, and Substance Involvement Screening Test).

**RESULTS:**

Out of the 858 students who participated, 71 (8.3%) were victims of sexual violence, 52 girls (73.2%). In the victims of violence group there were more students who already had the first sexual intercourse (p = 0.029), students who already had become pregnant (p = 0.001), students with higher scores for depressive (p < 0.001) and anxious symptoms (p = 0.001), students with worse quality of life (p < 0.001), and who used more tobacco (p = 0.008) and marijuana (p = 0.025) as well as abused hypnotics or sedatives (p = 0.048) than in the non-victim group.

**CONCLUSION:**

The abuses are presented in several forms and affect, even in long term, the survivors' life. The sexual violence theme should be addressed and widely discussed in all spheres of society in order to mobilize, to sensitize, and provide society with knowledge, demystifying this subject and drawing attention to this important social issue.

## INTRODUCTION

The literature presents several definitions and terms used to characterize sexual violence, such as rape, sexual abuse, indecent assault, sexual harassment, and obscene acts ^1^^–^^5^ . The use of a broad definition favors survivors of this crime, because it no longer considers trivial acts that, until then, could seem acceptable and commonplace, but that bring great harm to victims.

The term sexual violence characterizes sexual touch, the attempt to obtain forced sex, situation in which someone is physically forced to have sexual intercourse against their will or forced to do something in the sexual context that the individual considers as humiliating or degrading ^3^ . Sexual violence does not imply the penetration of penis into the vagina or anus, therefore men can also be victims of this type of violence.

The World Health Organization (WHO) defines the term “sexual violence” against children as their involvement in sexual activity that they do not fully comprehend and do not have the ability to consent or even activities that violate social laws or regulations ^6^ . The exploratory use of children in pornographic performances and materials, acts of sexual nature that do not involve contact (such as voyeurism or sexual harassment), and online exploitation complete WHO's definition ^6^ . The term “sexual violence” will be used in this work, using these comprehensive concepts.

This is a universal phenomenon, occurring in any sex, age, race or social class ^3^^,^^6^^,^^7^ . Women are more exposed to this type of aggression, whether as a child, adolescent, young adult or adult woman ^3^^,^^7^^,^^8^ . It is estimated that 7.0% of women will be victims of sexual violence, not perpetrated by intimate partners, throughout their lives ^8^ . The statistics of sexual violence perpetrated against children and adolescents highlight this type of violence as a major social problem ^6^^,^^7^^,^^8^ . The prevalence of sexual abuse in childhood is around 8.0% for boys and 18.0% for girls, and the latter are more likely to be victim from physical and sexual violence performed by intimate partners, such as rape, early and forced marriage, and sexual exploitation ^8^^,^^9^ .

In Brazil, the lack of systematized and continuous information hampers the dimensioning and cope with the problem. A recent study, based on reported information, showed 2,010 records of sexual abuse among adolescents and adult women between 2008 and 2013 in the state of Santa Catarina, in Southern Brazil, that is, 335 cases of abuse per year, 28 cases per month or almost one case per day ^10^ . Although the *Estatuto da Criança e do Adolescente* (ECA—Child and Adolescent Statute), published in 1990, established the mandatory notification of all cases of ill-treatment against children and adolescents, only in 2006 the Brazilian Ministry of Health implemented the *Sistema de Vigilância de Violências e Acidentes* (VIVA—Surveillance System for Violence and Accidents), aiming to collect data in a standardized manner, allowing the regular analysis of this information ^11^ . However, it is known that this type of transgression is often neglected, often occurs in a veiled manner and, in most cases, it is not reported. Therefore, the incidence rates are probably higher than those published, as the results obtained in another publication based on the notifications, in Santa Catarina, almost in the same period, but referring to the violation committed against children and adolescents : 477 suspected or confirmed cases of sexual abuse were recorded between 2008 and 2014, i.e., about 68 cases per year or approximately six reported cases per month–rates much lower than those found in the aforementioned study, indicating that this type of crime is underreported when perpetrated in childhood or adolescence ^6^^,^^12^ .

It is known that sexual violence harms the health of victims, with numerous physical and psychological consequences such as depression, anxiety, substance abuse, eating disorders, sleep disorders, sexual dysfunction, post-traumatic stress disorder (PTSD), sexually transmitted infections (STI) and suicidal ideation ^2^^,^^3^^,^^13^ . Publications struggle to bring answers about sexual violence to broaden the knowledge of what is happening to the victims, trying to prevent or to minimize the damage caused by this atrocity and promote a better confrontation ^6^^,^^9^^,^^10^^,^^12^ . Most studies research populations that are or were under follow-up in services that attend victims of sexual violence, individuals who went for support or reported the occurrence ^5^^,^^10^^,^^12^ .

This study aims to investigate the prevalence of sexual violence in a population of adolescents and young adults, individuals who were neither inserted in care services for victims of violence, nor necessarily reported the violation, as well as to know the presence of depressive and anxious symptoms, quality of life and tobacco, alcohol and substances use by this population and compare it with a population that has not been abused.

## METHODS

This is a cross-sectional, descriptive, and observational study carried out in a non-representative, selective sample of students, both male and female, from a public university in São Paulo. All students under 25 years of age enrolled in the institution who were in the classroom at the time of the research and agreed to participate in the study by reading and signing the informed consent form (ICF) for those aged over 18 years. Those students younger than 18 years had to read and to sign the consent form; their guardians also provided ICF. Therefore, participants were recruited between 2016 and 2017 and invited to answer profile and behavior questionnaires that characterized them according to age, gender (male and female), sexual behavior (age of the first sexual intercourse and the presence of pregnancy), and socioeconomic level (classes A1, A2, B1, B2, C1, C2, D and E) according to the classification of the *Associação Brasileira de Empresas de Pesquisa* (ABEP—Brazilian Association of Research Companies) ^14^ . Furthermore, complemental instruments were used, assessing the presence of physical and sexual abuse and neglect in the family (Questionnaire on Exposure to Traumatizing Events – QUESI) ^15^ and quality of life (World Health Organization Quality of Life Assessment–WHOQOL), ^16^ instruments that track depressive (Beck Depression Inventory—BDI) ^17^ and anxious symptoms (Beck Anxiety Inventory—BAI), ^17^ and screen for involvement with smoking, alcohol and other drugs (Alcohol, Smoking and Substance Involvement Screening Test—ASSIST) ^18^ . Regarding the characterization of the sample, individuals aged between 10 years and 20 incomplete years were considered as adolescents, and those aged between 20 and 24 years as young adults, according to WHO's delimitations on adolescence and youth ^19^ .

Statistical analysis of the collected information was initially carried out by mean, median, minimum and maximum values, standard deviation, and absolute and relative frequencies (percentage). Pearson's chi-square test, Fisher's exact or its extension, and Mann-Whitney or t-Student for independent samples were used to confirm or refute evidence found in the descriptive analysis. The level of alpha significance ≤ 5% was used in all outcomes obtained by the inferential analyses.

This study is in accordance with the Resolution No. 466/12 of the National Health Council of the Brazilian Ministry of Health, and it was approved by the Research Ethics Committee of the Universidade Federal de São Paulo (UNIFESP), project No. 0826/2016, under opinion No. 2,317,772.

## RESULTS

Out of the 1,308 students enrolled, 1,056 students were at the university facilities, out of whom 17 refused to participate, 175 were excluded because they were 25 years or older (24 years was the age limit for participation in the study, because adolescents and young adults were determined as target sample) and six students were excluded for not answering a large part of the questionnaires, totaling 858 participants. The 71 students who indicated any response other than “never” in items 21, 22, 23, 24, and 27 of QUESI—whose answers possibilities were “never,” “few times,” “sometimes,” “often,” and “always”—were considered victims of sexual violence. The questions were: 21. “They tried to touch me or made me touch them in a sexual manner”; 22. “They threatened to hurt me or to tell lies about me if I did not do something sexual”; 23. “They tried to force me to do something sexual or watch things about sex”; 24. “Someone molested me”; and 27. “I believe I have been sexually abused.”

Most of the 858 students assessed were female (n = 549; 64.0%), with a mean age of 21.1 years, ranging between 17 and 24 years. Regarding socioeconomic status, 369 students were classified as economic class A (43.0%), 192 as class B1 (22.4%), 196 as B2 (22.8%), 60 as class C1 (7.0%), 11 as class C2 (1.3%), and one as class D/E (0.1%). In total, 29 students did not answer this question. Most participants belonged to socioeconomic status up to class B2 (88.2%).

**Figure 1:**
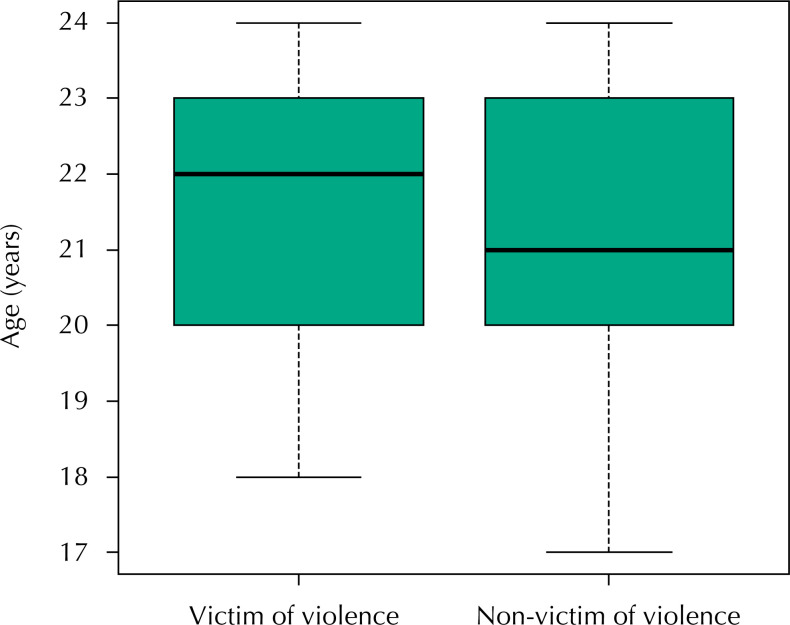
Boxplot of the age of victims and non-victims of sexual violence.

**Figure 2:**
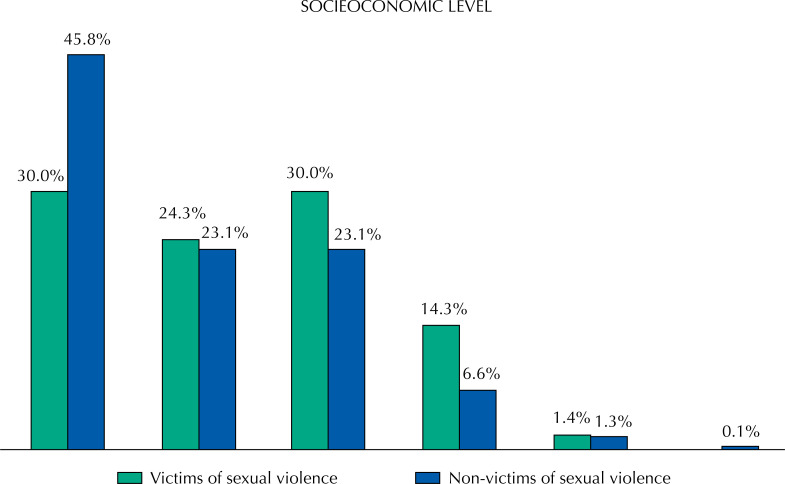
Distribution of victims and non-victims of sexual violence according to socioeconomic level.

Regarding the occurrence of sexual violence at some point in their lives, 71 students (8.3%, with 95% confidence interval between 6.4% and 10.1%) were victims of sexual violence, 73.2% girls (n = 52) and 26.8% boys (n = 19). The victims of violence presented higher age (p = 0.014) and lower socioeconomic status (p = 0.003), i.e., there were more victims in classes B2 and C, proportionally ( Figures 1 and 2 ).

A total of 60 students (84.5%) from the victim of violence group had already had the first sexual intercourse and 11 (15.5%) students have not had the first sexual intercourse. A significant difference was obtained for this variable (p = 0.029). In total, five (12.0%) students of the victim group and four (1.2%) of the non-victim of sexual violence had already become pregnant (p = 0.001). The relationship between the first sexual intercourse and the gender of the students was investigated ( Table 1 ). The first sexual intercourse was more frequent among male students than among the female students as a whole and when only those who were not victims of violence were evaluated. In the victim of violence group, the occurrence of the first sexual intercourse was similar between genders ( Table 1 ).

**Table 1:** Distribution of occurrence of the first sexual intercourse according to gender (female and male) and if it was a sexual violence or not.

		First sexual intercourse				Total		
	Gender	Yes		No				p
		n	%	n	Frequency	n	%	
Victim	Female	42	80.8	10	19.2	52	100.0	0.267 a
	Male	18	94.7	1	5.3	19	100.0	
	Total	60	84.5	11	15.5	71	100.0	
Non-victim	Female	334	67.6	160	32.4	494	100.0	< 0.001 b
	Male	232	81.1	54	18.9	286	100.0	
	Total	566	72.6	214	27.4	780	100.0	
Total	Female	376	68.9	170	31.1	546	100.0	< 0.001 b
	Male	250	82.0	55	18.0	305	100.0	
	Total	626	73.6	225	26.4	851	100.0	

a Fisher's Exact Test.

b Pearson's Chi-square.

The presence of symptoms of depression was compared using the BDI instrument; for anxious symptoms BAI was used; and for quality of life the WHOQOL was used between the victim of sexual violence and non-victim groups ( Table 2 ). It was observed that students who were victims of sexual violence presented had higher scores for depressive (p < 0.001) and anxious (p = 0.001) symptoms, as well as worse quality of life (p < 0.001) than non-victims.

**Table 2:** Measurements of the scores of depression (BDI), anxiety (BAI), and quality of life (WHOQOL) questionnaires according to having been a victim of sexual violence or not.

Instruments	Measurement	Victim of violence	Non-victim of violence	Total	p
BDI	n	69	762	831	< 0.001 *
	Mean (SD)	13.4 (8.4)	9.2 (7.3)	9.6 (7.4)	
	Median	13.0	7.0	8.0	
	Minimum–Maximum	1–35	0–37	0–37	
BAI	n	71	783	854	0.001 *
	Mean (SD)	12.1 (9.0)	8.7 (7.8)	9.0 (7.9)	
	Median	9.0	7.0	7.0	
	Minimum–Maximum	0–35	0–47	0–47	
WHOQOL					
	n	71	781	852	< 0.001 *
	Mean (SD)	59.5 (14.6)	67.2 (13.2)	66.6 (13.5)	
	Median	58.7	68.3	67.3	
	Minimum–Maximum	28.9–87.5	24.1–100.0	24.1–100.0	

n: total number of occurrences; SD: standard deviation; BDI: Beck Depression Inventory; BAI: Beck Anxiety Inventory; WHOQOL: World Health Organization's Quality of Life Assessment

* Mann-Whitney

This research also studied the comparison between the use of tobacco, alcohol, and substances between the victim and non-victim of sexual violence groups, using the ASSIST instrument. According to inferential results, there are more victims of sexual violence among students who abusively consumed tobacco (p = 0.008), marijuana (p = 0.025), and hypnotics or sedatives (p = 0.048) as shown in Table 3 . There was no difference between the groups for the other drugs. Note that, because of the low use of opioids in the groups, it was not possible to perform a statistical comparison for this type of drug.

**Table 3:** Distribution of use of alcohol, tobacco, and other drugs among students, according to having been a victim of sexual violence or not.

		Victim		Non-victim		Total		p
		n	%	n	%	n	%	
Alcoholic beverages	Occasional use	28	40.6	389	50.1	417	49.3	0.128 a
	Abuse	41	59.4	387	49.9	428	50.7	
	Addiction	-	-	-	-	-	-	
	Total	69	100.0	776	100.0	845	100.0	
Tobacco	Occasional use	57	81.4	700	90.3	757	89.6	0.008 b
	Abuse	12	17.2	75	9.7	87	10.3	
	Addiction	1	1.4	-	-	1	0.1	
	Total	70	100.0	775	100.0	845	100.0	
Marijuana	Occasional use	58	84.0	702	90.6	760	90.0	0.025 b
	Abuse	10	14.5	73	9.4	83	9.8	
	Addiction	1	1.5	-	-	1	0.2	
	Total	69	100.0	775	100.0	844	100.0	
Cocaine/crack	Occasional use	70	100.0	768	99.1	838	99.2	> 0.999 b
	Abuse	-	-	7	0.9	7	0.8	
	Addiction	-	-	-	-	-	-	
	Total	70	100.0	775	100.0	845	100.0	
Psychostimulants	Occasional use	68	97.1	758	97.6	826	97.5	0.690 b
	Abuse	2	2.9	19	2.4	21	2.5	
	Addiction	-	-	-	-	-	-	
	Total	70	100.0	777	100.0	847	100.0	
Inhalants	Occasional use	68	97.1	761	98.0	829	97.9	0.655 b
	Abuse	2	2.9	16	2.0	18	2.1	
	Addiction	-	-	-	-	-	-	
	Total	70	100.0	777	100.0	847	100.0	
Hypnotics/sedatives	Occasional use	65	92.9	756	97.4	821	97.0	0.048 b
	Abuse	5	7.1	20	2.6	25	3.0	
	Addiction	-	-	-	-	-	-	
	Total	70	100.0	776	100.0	846	100.0	
Hallucinogenic drugs	Occasional use	70	100.0	768	98.8	838	98.9	> 0.999 b
	Abuse	-	-	9	1.2	9	1.1	
	Addiction	-	-	-	-	-	-	
	Total	70	100.0	777	100.0	847	100.0	
Opioids	Occasional use	69	100.0	775	91.8	844	100.0	-
	Abuse	-	-	-	-	-	-	
	Addiction	-	-	-	-	-	-	
	Total	69	100.0	775	91.8	844	100.0	

a Pearson's Chi-square.

b Fisher's exact or its extent.

## DISCUSSION

This study used an intentional sample of undergraduate students, avoiding a long distance between the violence experienced and the survey, in order to not impair the memories about the events experienced. Also, the population was not composed of a large number of underage subjects, whose parents or guardians would have to consent the individuals' participation in the research; which could be an impairment, considering the delicacy of the theme. In this study, only 13 individuals were younger than 18 years, none of them victims of sexual violence, which facilitated the obtaining of the informed consent form. Considering that this study was carried out exclusively with undergraduate students ensured the absence of individuals with intellectual impairment, a situation that could hinder or impair proper filling of the instruments. Another reason to conduct the research with undergraduate students was avoiding individuals who were attending to an outpatient clinics to accompany victims of sexual violence.

International studies, as we present in the next paragraphs, indicate that sexual violence is a worldwide phenomenon. The prevalence found in literature are difficult to compare, since each study use different methodologies, presenting no pattern in victims' age groups nor in what is considered sexual violence. Despite the difficulty in comparing the prevalence, it is understood that any prevalence obtained in scientific studies should be considered alarming and relevant considering the atrocity represented by this type of violence.

In the United States, child sexual abuse is the third most common cause of violence against children, after neglect and physical abuse ^20^ . National data from the 13th Public Security Yearbook indicate that in 2018, 66,041 cases of rape were recorded in Brazil (average of 180 cases/day); 80.0% of the victims were female and 53.8% were under 13 years of age, i.e., four girls up to 13 years were raped per hour in 2018 ^21^ .

In this study, 8.3% of the students reported having suffered sexual violence at some point in childhood or adolescence, 73.2% of them were girls and 26.8% were boys. It is believed that these numbers may be underestimated, considering the refusal of some students to participate in this research. In agreement with what was found in this research, studies show that sexual violence is predominant in women ^3^^,^^21^^,^^22^ . It is believed that the underreporting among men is very expressive ^23^^,^^24^ . Social norms about masculinity can influence the boys' decision to tell their stories, fearing being considered as homosexuals ^23^^,^^24^ . Boys who are victim of abuse find themselves in a position of great fragility, contrasting with the dominant construct of masculinity imposed by society ^23^^,^^24^ .

In the studied sample was observed a predominance of socioeconomic classes A, B1, and B2, highlighting that, although the existence of a quota policy for admission to public universities since 2012, there is still an important difference in the access of the less favored classes. Although victims of violence were present in all social classes and only one individual belonging to class D/E participated in this study, there was a strong predominance of sexual violence in classes with lower purchasing power. Based on our outcomes, one can think about poverty factors that would place these individuals in a situation of greater risk and less protection. Carneiro and Veiga ^25^ say that poverty exposes individuals to risk situations, especially in contexts in which a social support network, which offers tools to cope with adversities, cannot be found. It is not possible to affirm that socioeconomic status is a direct risk factor for sexual violence, since, according to the literature, sexual violence affects all social classes equally, but it can be reflected that families with lack of financial resources may be more likely to leave their children at risk, neglecting care to them, often for lack of better options. A review of the literature published in 2018 states that female children in families whose incomes are below or on the poverty line are at greater risk of being victims, and that the parents' schooling level contributes to this risk, which is lower in families whose mothers present more than 12 years of schooling ^26^ . The absence of places in day care centers, full-time public schools, and free places that can receive children or adolescents in the period after school, a sad Brazilian reality, oblige less favored families, who do not have sufficient financial resources to pay for these services, to leave their children under the care of neighbors and family members with few conditions to supervise properly, or even alone at home or under the care of older siblings. Thus, children are exposed to risk situations often identified by the perpetrator, who is usually someone from the victim's social circle. The cruel person sees the lack of security surrounding the victim.

Notably, the students who were victims of sexual violence were older than the non-victims, that is, these individuals started their undergraduate course later. Probably, as these individuals are economically less favored, they come from more popular public or private schools, whose teaching is not enough for admission to very popular universities—requiring years of preparatory classes to be able to enter the desired courses. It is known that schools whose teaching is adequate and prepares students for admission to public universities in Brazil are expensive. On the other hand, sexual violence can greatly affect the victim's academic life ^27^ . Changes in cognitive development, language, memory, and school performance, in addition to lowering the perception of one's own performance and capacity have often been reported ^27^^,^^28^ . Although all the subjects of this research are undergraduate students, it can be reflected that the school damage resulting from abuse, forced victims of sexual violence to take more years of preparatory courses to enter the university, which had repercussions on the age of entry of these young people into higher education.

The relationship between the occurrence of sexual violence in childhood and adolescence and sexual behavior must be highlighted. More students who had already had the first sexual intercourse and students who had already become pregnant were found in the group victim of violence, suggesting that girls who were victims of violence are more sexually active compared to non-victims, corroborating with the literature on the experience of sexual abuse and development of risky sexual behavior, such as unprotected sexual activity, precocious, multiple partners, and promiscuity ^29^^,^^30^ . Studies have shown a relationship between sexual abuse in childhood in women with early first sexual intercourse, greater number of partners, increased risk of use of psychoactive substances before sexual practice, a higher number of unprotected relationships, and a higher prevalence of STI ^29^^,^^30^ . There is also evidence of increased risky sexual behaviors in male individuals who have been abused and have homosexual relationships, such as unprotected anal intercourse ^24^ .

When comparing the presence of depressive and anxious symptoms, quality of life and use of alcohol, tobacco, and other substances among the population victim of violence with the population that was not violated, results were found compatible with those in the literature. Survivors of sexual violence presented higher scores for depression, anxiety and worse quality of life when compared to those who were not victims violence. Regarding the use of legal or illegal drugs, individuals who were victims of abuse presented higher consumption of tobacco and marijuana and abused sedatives or hypnotics, perhaps to minimize the discomfort of depressive or anxious symptoms. It is well described that sexual violence is a major risk factor for the development of depression, anxiety, PTSD, sleep disorders, phobias, eating disorders, suicidal attempt or ideation, self-mutilation and drug abuse, with depression and PTSD remaining as the most common forms of mental disorders associated with sexual abuse ^1^^,^^3^ . When sexual violence affects children and adolescents, it becomes especially dramatic, since it threatens the sexual and psychic development of an individual in formation ^4^^,^^6^ . Children who are victims of sexual violence are more subject to tobacco and alcohol use or drug abuse, risky sexual behavior (early onset of consensual sexual activity, multiple partners, and unprotected sexual intercourse), shyness, isolation, vulnerability to becoming victims again of this or other forms of violence, academic problems, delinquency, low self-esteem, aggressiveness, self-destructive behaviors, hopelessness about the future, lies, difficulty in trusting others, and damage to their quality of life ^1^^–^^3^^,^^6^^,^^9^ .

It is important to consider that many of the violations experienced by the research subjects may have occurred in childhood or adolescence, periods somewhat distant from the moment of this research. However, it can be seen that negative consequences of abuse can persist for a long time. Notably, this population was not recruited in support services for victims of violence, which could be a bias in the results presented, since individuals usually seek and are monitored in these services because they need support, that is, because they are, in a way, more fragile and prone to being depressed, with anxious symptoms and with a feeling of worse quality of life or predisposed to use more alcohol and other drugs.

Considering the results obtained here, the main objective of all public health programs should be the prevention of sexual violence against children and adolescents. This type of brutality should shock society and be treated as a heinous crime. Then, this violence would not be tolerated and, if it occurs, it would be promptly denounced.

Due to the burden this type of violence entails for survivors, health systems, and society as a whole, it should be a matter of great scientific and popular interest. It is believed that education and production of material on the subject can mobilize, sensitize, and provide parents, children, peers, educators, health professionals, and the whole collective with knowledge, demystifying the subject and drawing attention to this important social issue.

It is pertinent to draw attention to the fact that all participants of the research are individuals who are regularly enrolled in a public university in Brazil, an institution with fierce competition for admission, that is, they are admittedly capable and intelligent subjects, who, despite the dramas experienced, are moving forward.

This work indicated the need to give voice to the subjects who were violated, in order to know their life histories, their anguish, their ways, their overcoming. This study is still being carried out, using a qualitative approach, by obtaining oral history of individuals who were victims of sexual abuse.
