# Supplementary material for: The effects of sexual violence experienced in childhood and adolescence on undergraduate students
Source: Rev Saude Publica. 2020 Nov 27;54:134. doi: 10.11606/s1518-8787.2020054002576 (PMC7703533; doi:10.11606/s1518-8787.2020054002576)
Supplement: Translation

# Translation: Os impactos da violência sexual vivida na infância e adolescência em universitários

**Authors:** Flávia Calanca da Silva, Aline Monge, Carlos Alberto Landi, Gabriel Amaral Zenardi, Denise Chrysostomo Suzuki, Maria Sylvia de Souza Vitalle

**Affiliations:** I São PauloSP Brasil Universidade Federal de São Paulo. Escola Paulista de Medicina. Departamento de Pediatria. São Paulo, SP, Brasil; II São PauloSP Brasil Universidade Federal de São Paulo. Escola Paulista de Medicina. Programa de Pós-Graduação em Saúde Coletiva. São Paulo, SP, Brasil; III GuarulhosSP Brasil Universidade Federal de São Paulo. Escola de Filosofia, Letras e Ciências Humanas. Programa de Pós-Graduação em Educação e Saúde. Guarulhos, SP, Brasil; IV São PauloSP Brasil Faculdade de Ciências Médicas da Santa Casa de São Paulo. Departamento de Atenção Primária. São Paulo, SP, Brasil; V São PauloSP Brasil Universidade Nove de Julho. Faculdade de Medicina. Módulo de Pediatria. São Paulo, SP, Brasil; VI São PauloSP Brasil Universidade Federal de São Paulo. Escola Paulista de Medicina. São Paulo, SP, Brasil

**Keywords:** Adolescente, Adulto Jovem, Comportamento Sexual, Abuso Sexual na Infância, Delitos Sexuais, Transtornos Mentais, epidemiologia, Transtornos Relacionados ao Uso de Substâncias

## Abstract

**OBJETIVO:**

Investigar a prevalência de adolescentes e adultos jovens que foram vítimas de violência sexual em algum momento da vida e comparar a presença de sintomas depressivos e ansiosos, qualidade de vida e uso de álcool, tabaco e drogas ilícitas entre esta população e a que não sofreu abuso.

**MÉTODOS:**

Aplicaram-se questionários e instrumentos validados, em uma população de estudantes universitários, para avaliar: perfil e comportamento sexual, nível socioeconômico, presença ou não de violência sexual (Questionário sobre a Exposição a Eventos Traumatizantes), sintomas depressivos (Inventário de Depressão de Beck) e ansiosos (Inventário de Ansiedade de Beck), qualidade de vida (World Health Organization's Quality of Life Assessment) e o uso ou abuso de tabaco, álcool e drogas ilícitas (Teste para Triagem do Envolvimento com Fumo, Álcool e Outras Drogas).

**RESULTADOS:**

Dos 858 alunos que responderam à pesquisa, 71 (8,3%) foram vítimas de violência sexual, sendo 52 meninas (73,2%). No grupo vítima de abuso havia mais alunos que já tinham tido a coitarca (p = 0,029), alunas que já engravidaram (p = 0,001), estudantes com maiores escores para sintomas depressivos (p < 0,001) e ansiosos (p = 0,001), alunos com pior qualidade de vida (p < 0,001) e que usavam mais tabaco (p = 0,008) e maconha (p = 0,025) bem como abusavam de hipnóticos ou sedativos (p = 0,048) que no grupo não vítima.

**CONCLUSÃO:**

Os impactos causados pelo abuso são diversos e afetam, mesmo no longo prazo, a vida dos sobreviventes. Abordar o tema e o discutir, amplamente, em todas as esferas da sociedade é uma forma de mobilizar, sensibilizar e instrumentalizar o coletivo, desmistificando o assunto e chamando atenção para essa importante questão social.

## INTRODUÇÃO

Há na literatura inúmeras definições e termos utilizados para caracterizar a violência do tipo sexual, como estupro, abuso sexual, atentado violento ao pudor, assédio sexual e atos obscenos ^1^^–^^5^ . A utilização de definição ampla favorece os sobreviventes deste tipo de crime, pois deixa de considerar banais atos que, até então, podiam parecer aceitáveis e corriqueiros, mas que trazem grandes prejuízos às vítimas.

O termo violência sexual caracteriza o toque sexual, a tentativa de obter sexo e o sexo forçado ou sob pressão, situações nas quais se é fisicamente forçado a ter intercurso sexual quando não se quer ou se é forçado a fazer algo no contexto sexual que é, pelo indivíduo, considerado humilhante ou degradante ^3^ . Não implica apenas a penetração do pênis na vagina ou ânus e, por isso, homens também podem ser vítimas desse tipo de violência.

A Organização Mundial de Saúde (OMS) define o termo “violência sexual” contra a criança como o envolvimento dela em atividade sexual que ela não compreende completamente e não tem habilidade para dar consentimento ou que viola as leis ou normas sociais ^6^ . O uso exploratório de crianças em performances e materiais pornográficos, atos de natureza sexual que não envolvem contato (tais como voyeurismo ou assédio sexual) e exploração on-line completam essa definição ^6^ . Utilizando esses conceitos abrangentes, o termo “violência sexual” será utilizado neste trabalho.

Trata-se de fenômeno universal, que pode ocorrer em qualquer sexo, idade, raça ou classe social ^3^^,^^6^^,^^7^ . O sexo feminino é mais exposto a esse tipo de agressão, seja quando criança, adolescente, jovem ou mulher adulta ^3^^,^^7^^,^^8^ . Estima-se que 7,0% das mulheres serão vítimas de violência sexual, não perpetrada por parceiros íntimos, ao longo de suas vidas ^8^ . Quando se atenta às estatísticas de violência sexual perpetrada contra crianças e adolescentes, observa-se que é um grande problema social ^6^^,^^7^^,^^8^ . A prevalência de abuso sexual na infância está em torno de 8,0% para meninos e 18,0% para meninas, tendo estas maior probabilidade de sofrer violência física e sexual praticada por parceiros íntimos, estupro, casamento precoce e forçado e exploração sexual ^8^^,^^9^ .

No Brasil, a escassez de informações sistematizadas e contínuas dificulta o dimensionamento e enfrentamento do problema. Um recente trabalho publicado, a partir de informações notificadas, evidenciou 2.010 registros de abuso sexual entre adolescentes e mulheres adultas nos anos de 2008 a 2013 no estado de Santa Catarina, na região Sul do Brasil, ou seja, 335 casos de abuso por ano, 28 casos por mês ou quase um caso novo por dia ^10^ . Apesar de o Estatuto da Criança e do Adolescente, publicado em 1990, estabelecer a obrigatoriedade de notificação de todos os casos de maus-tratos contra crianças e adolescentes, somente em 2006 o Ministério da Saúde implantou, nacionalmente, o Sistema de Vigilância de Violências e Acidentes (Viva), com o objetivo de coletar dados de forma padronizada, permitindo a análise regular dessas informações ^11^ . Sabe-se, porém, que esse tipo de transgressão é muitas vezes negligenciada, ocorre de maneira velada e, na maioria das vezes, não é delatada, levando a crer que as taxas são bem maiores do que as publicadas, como mostram os resultados obtidos em outra publicação com base nas notificações realizadas também em Santa Catarina, quase no mesmo período, porém referentes à violação cometida contra crianças e adolescentes: 477 casos suspeitos ou confirmados de abuso sexual foram registrados entre os anos de 2008 e 2014, ou seja, cerca de 68 casos ao ano ou, aproximadamente, seis casos notificados por mês – índices muito menores do que os encontrados no trabalho citado anteriormente, apontando o quão subnotificado é esse tipo de crime quando perpetrado na infância ou adolescência ^6^^,^^12^ .

Sabe-se que a violência sexual acarreta danos para saúde das vítimas, com inúmeras consequências físicas e psicológicas como depressão, ansiedade, abuso de substâncias, transtorno alimentar, distúrbio do sono, disfunção sexual, transtorno de estresse pós-traumático (TEPT), infecções sexualmente transmissíveis (IST) e ideação suicidas ^2^^,^^3^^,^^13^ . Publicações buscam trazer respostas sobre o assunto para ampliar o entendimento do que se passa com as vítimas, tentando prevenir ou minimizar os danos causados por essa atrocidade e promover um melhor enfrentamento ^6^^,^^9^^,^^10^^,^^12^ . Grande parte dos trabalhos estudam populações que se encontram ou se encontravam em acompanhamento em serviços de atendimento a vítimas de violência sexual, indivíduos que foram em busca de apoio ou notificaram a ocorrência ^5^^,^^10^^,^^12^ .

Objetiva-se, com a realização deste trabalho, investigar a prevalência de violência sexual em uma população de adolescentes e adultos jovens universitários, sujeitos que não se encontravam necessariamente inseridos em serviços de atendimento à vítimas de violência, nem obrigatoriamente realizaram a notificação da violação, bem como conhecer a presença de sintomas depressivos e ansiosos, a qualidade de vida e o uso de tabaco, álcool e outras drogas por essa população e comparar com uma população que não foi vítima de abuso.

## MÉTODOS

Estudo transversal, descritivo e observacional, realizado em amostra não representativa, seletiva, de população de estudantes, dos sexos masculino e feminino, de uma universidade pública de São Paulo. Todos os estudantes menores de 25 anos matriculados na instituição que estavam em sala de aula no momento da realização da pesquisa e que aceitaram participar do estudo mediante leitura e assinatura do termo de consentimento livre e esclarecido (TCLE) para os maiores de 18 anos, ou leitura e assinatura do termo de assentimento (TA) pelo aluno juntamente com a leitura e assinatura do TCLE pelo responsável para os menores de 18 anos, foram recrutados durante os anos de 2016 e 2017 e convidados a responder questionários de perfil e comportamento que os caracterizaram segundo idade, sexo (masculino e feminino), comportamento sexual (idade da coitarca e presença de gravidez) e nível socioeconômico (classes A1, A2, B1, B2, C1, C2, D e E) de acordo com classificação da Associação Brasileira de Empresas de Pesquisa (ABEP) ^14^ , além de instrumentos que avaliam a presença de abuso físico, sexual e negligência na família (Questionário sobre a Exposição a Eventos Traumatizantes – Quesi) ^15^ e a qualidade de vida (World Health Organization's Quality of Life Assessment – WHOQOL) ^16^ , rastreiam sintomas depressivos (Inventário de Depressão de Beck – BDI) ^17^ e ansiosos (Inventário de Ansiedade de Beck – BAI) ^17^ e triam o envolvimento com fumo, álcool e outras drogas (Teste para Triagem do Envolvimento com Fumo, Álcool e Outras Drogas – ASSIST) ^18^ . Sobre a caracterização do público-alvo, considerou-se como adolescentes os indivíduos com idade de 10 a 20 anos incompletos, e como jovens adultos aqueles dos 20 a 24 anos, de acordo com as delimitações da OMS sobre adolescência e juventude ^19^ .

A análise estatística das informações coletadas foi inicialmente feita de forma descritiva por meio da média, mediana, valores mínimo e máximo, desvio-padrão e frequências absoluta e relativa (porcentagem). As análises inferenciais empregadas, com o intuito de confirmar ou refutar evidências encontradas na análise descritiva, foram o teste do qui-quadrado de Pearson, exato de Fisher ou sua extensão e Mann-Whitney ou t-Student para amostras independentes. Em todas as conclusões obtidas pelas análises inferenciais, foi utilizado o nível de significância alfa menor ou igual a 5%.

O estudo está de acordo com a Resolução n° 466/12 do Conselho Nacional de Saúde do Ministério da Saúde, tendo sido aprovada pelo Comitê de Ética em Pesquisa da Universidade Federal de São Paulo (Unifesp), projeto n° 0826/2016, sob parecer n° 2.317.772.

## RESULTADOS

Dos 1.308 estudantes matriculados, estavam em sala de aula 1.056 alunos, dos quais 17 se recusaram a participar, 175 foram excluídos por terem 25 anos ou mais (24 anos foi a idade-limite para participação no estudo, pois se determinou adolescentes e adultos jovens como público-alvo) e 6 alunos foram excluídos por não responderem grande parte dos questionários, totalizando 858 participantes. Os 71 estudantes que assinalaram qualquer resposta diferente de “nunca” nos itens 21, 22, 23, 24 e 27 do Quesi – cujas possibilidades de resposta eram “nunca”, “poucas vezes”, “às vezes”, “muitas vezes” e “sempre” – foram considerados vítimas de violência sexual. As perguntas foram: 21. “Tentaram me tocar ou me fizeram tocar de uma maneira sexual”, 22. “Ameaçaram me machucar ou contar mentiras sobre mim se eu não fizesse algo sexual”, 23. “Tentaram me forçar a fazer algo sexual ou assistir coisas sobre sexo”, 24. “Alguém me molestou” e 27. “Eu acredito que fui abusado(a) sexualmente”.

Dos 858 alunos avaliados, a maioria era do sexo feminino (n = 549; 64,0%), com idade média de 21,1 anos, variando de 17 a 24 anos. Em relação ao nível socioeconômico, encontraram-se 369 alunos na classe econômica A (43,0%), 192 na classe B1 (22,4%), 196 na B2 (22,8%), 60 na classe C1 (7,0%), 11 na classe C2 (1,3%) e 1 na classe D/E (0,1%). Não responderam a essa questão 29 estudantes. A maioria pertencia ao nível socioeconômico até classe B2 (88,2%).

Em relação à ocorrência de violência sexual em algum momento de suas vidas, 71 alunos (8,3%, com intervalo de confiança de 95% de 6,4% a 10,1%) foram vítimas de violência sexual, sendo 73,2% moças (n = 52) e 26,8% rapazes (n = 19). As vítimas de violência apresentaram maior idade (p = 0,014) e menor nível socioeconômico (p = 0,003), ou seja, havia mais alunos vítimas nas classes B2 e C, proporcionalmente ( Figuras 1 e 2 ).

**Figura 1:** Boxplot da idade dos alunos vítimas e não vítimas de violência sexual.

**Figura 2:** Distribuição dos alunos vítimas e não vítimas de violência sexual segundo nível socioeconômico.

Sessenta alunos (84,5%) do grupo vítima de violência já haviam tido a coitarca e 11 (15,5%) estudantes ainda não. Obteve-se uma diferença significativa para essa variável (p = 0,029). Cinco (12,0%) universitárias do grupo vítima e quatro (1,2%) do grupo não vítima de violência sexual já tinham engravidado (p = 0,001). Foi investigada a relação entre coitarca e sexo dos alunos ( Tabela 1 ). A coitarca foi mais frequente entre os alunos do sexo masculino que entre as alunas no grupo como um todo e quando avaliados somente os que não foram vítimas de violência. No grupo vítima de violência, a ocorrência de coitarca foi semelhante entre os sexos ( Tabela 1 ).

**Tabela 1:** Distribuição de ocorrência da coitarca segundo sexo (feminino e masculino) e ter sido ou não vítima de violência sexual.

	Sexo	Coitarca				Total		p
		Sim		Não				
		n	%	n	Frequência	n	%	
Vítima	Feminino	42	80,8	10	19,2	52	100,0	0,267 a
	Masculino	18	94,7	1	5,3	19	100,0	
	Total	60	84,5	11	15,5	71	100,0	
Não vítima	Feminino	334	67,6	160	32,4	494	100,0	< 0,001 b
	Masculino	232	81,1	54	18,9	286	100,0	
	Total	566	72,6	214	27,4	780	100,0	
Total	Feminino	376	68,9	170	31,1	546	100,0	< 0,001 b
	Masculino	250	82,0	55	18,0	305	100,0	
	Total	626	73,6	225	26,4	851	100,0	

a Exato de Fisher

b Qui-quadrado de Pearson

Comparou-se a presença de sintomas depressivos utilizando o instrumento BDI; sintomas ansiosos, do BAI; e qualidade de vida, do WHOQOL entre os grupos vítima de violência sexual e não vítima ( Tabela 2 ). Observou-se que os estudantes vítimas de violência sexual apresentavam maiores escores para sintomas depressivos (p < 0,001) e ansiosos (p = 0,001) bem como pior qualidade de vida (p < 0,001) do que os não vítimas.

**Tabela 2:** Medidas dos escores dos questionários de depressão (BDI), ansiedade (BAI) e qualidade de vida (WHOQOL) segundo ter sido vítima ou não de violência sexual.

Instrumentos	Medida	Vítima de violência	Não vítima de violência	Total	p
BDI	n	69	762	831	< 0,001 *
	Média (DP)	13,4 (8,4)	9,2 (7,3)	9,6 (7,4)	
	Mediana	13,0	7,0	8,0	
	Mínimo–máximo	1–35	0–37	0–37	
BAI	n	71	783	854	0,001 *
	Média (DP)	12,1 (9,0)	8,7 (7,8)	9,0 (7,9)	
	Mediana	9,0	7,0	7,0	
	Mínimo–máximo	0–35	0–47	0–47	
WHOQOL					
	n	71	781	852	< 0,001 *
	Média (DP)	59,5 (14,6)	67,2 (13,2)	66,6 (13,5)	
	Mediana	58,7	68,3	67,3	
	Mínimo–máximo	28,9–87,5	24,1–100,0	24,1–100,0	

n: número total de ocorrências; DP: desvio-padrão; BDI: *Beck Depression Inventory* ; BAI: *Beck Anxiety Inventory* ; WHOQOL: *World Health Organization's Quality of Life Assessment*

* Mann-Whitney

Foi ainda objeto de investigação dessa pesquisa a comparação do uso de tabaco, bebida alcoólica e outras drogas entre os grupos vítima e não vítima de violência sexual, utilizando o instrumento ASSIST. Conforme resultados inferenciais, há mais vítimas de violência sexual entre os alunos que faziam uso abusivo de tabaco (p = 0,008), maconha (p = 0,025) e hipnóticos ou sedativos (p = 0,048) como mostra a Tabela 3 . Não houve diferença entre os grupos para as demais drogas. Vale destacar que, pelo pouco uso de opioides nos grupos, não foi possível realizar a comparação estatística para esse tipo de droga.

**Tabela 3:** Distribuição do uso de álcool, tabaco e outras drogas entre os alunos, segundo ter sido ou não vítima de violência sexual.

		Vítima		Não vítima		Total		p
		n	%	n	%	n	%	
Bebida alcoólica	Uso ocasional	28	40,6	389	50,1	417	49,3	0,128 a
	Abuso	41	59,4	387	49,9	428	50,7	
	Dependência	-	-	-	-	-	-	
	Total	69	100,0	776	100,0	845	100,0	
Tabaco	Uso ocasional	57	81,4	700	90,3	757	89,6	0,008 b
	Abuso	12	17,2	75	9,7	87	10,3	
	Dependência	1	1,4	-	-	1	0,1	
	Total	70	100,0	775	100,0	845	100,0	
Maconha	Uso ocasional	58	84,0	702	90,6	760	90,0	0,025 b
	Abuso	10	14,5	73	9,4	83	9,8	
	Dependência	1	1,5	-	-	1	0,2	
	Total	69	100,0	775	100,0	844	100,0	
Cocaína/crack	Uso ocasional	70	100,0	768	99,1	838	99,2	> 0,999 b
	Abuso	-	-	7	0,9	7	0,8	
	Dependência	-	-	-	-	-	-	
	Total	70	100,0	775	100,0	845	100,0	
Estimulantes	Uso ocasional	68	97,1	758	97,6	826	97,5	0,690 b
	Abuso	2	2,9	19	2,4	21	2,5	
	Dependência	-	-	-	-	-	-	
	Total	70	100,0	777	100,0	847	100,0	
Inalantes	Uso ocasional	68	97,1	761	98,0	829	97,9	0,655 b
	Abuso	2	2,9	16	2,0	18	2,1	
	Dependência	-	-	-	-	-	-	
	Total	70	100,0	777	100,0	847	100,0	
Hipnóticos/sedativos	Uso ocasional	65	92,9	756	97,4	821	97,0	0,048 b
	Abuso	5	7,1	20	2,6	25	3,0	
	Dependência	-	-	-	-	-	-	
	Total	70	100,0	776	100,0	846	100,0	
Drogas alucinógenas	Uso ocasional	70	100,0	768	98,8	838	98,9	> 0,999 b
	Abuso	-	-	9	1,2	9	1,1	
	Dependência	-	-	-	-	-	-	
	Total	70	100,0	777	100,0	847	100,0	
Opioides	Uso ocasional	69	100,0	775	91,8	844	100,0	-
	Abuso	-	-	-	-	-	-	
	Dependência	-	-	-	-	-	-	
	Total	69	100,0	775	91,8	844	100,0	

a Qui-quadrado de Pearson

b Exato de Fisher ou sua extensão

## DISCUSSÃO

Este estudo utilizou uma amostra intencional de estudantes universitários, objetivando, com isso, que a violência vivida não estivesse temporalmente tão distante a ponto de prejudicar as memórias sobre os acontecimentos experimentados, tampouco a população fosse formada por grande número de sujeitos menores de idade, cujos pais ou responsáveis necessitassem consentir para com a participação na pesquisa; o que poderia ser um impedimento, tendo em vista a delicadeza do tema. Observaram-se nesta pesquisa apenas 13 indivíduos menores de 18 anos, nenhum deles vítima de violência sexual, o que de certa forma facilitou a obtenção do TCLE. Trabalhar com universitários garantiu ainda a ausência de sujeitos com déficit intelectual, situação que poderia dificultar ou prejudicar o preenchimento adequado dos instrumentos. Recrutar indivíduos fora de ambulatórios de acompanhamento às vítimas de violência sexual foi outra razão para realizar a pesquisa com estudantes em uma universidade.

Os estudos internacionais, como apresentamos nos próximos parágrafos, apontam que a violência sexual é um fenômeno mundial. As prevalências encontradas na literatura são de difícil comparação, visto que diferentes metodologias são utilizadas nos estudos, não havendo uniformidade nas faixas etárias avaliadas no recrutamento das vítimas nem no que é considerado violência sexual. Apesar da dificuldade em compará-las, entende-se que qualquer prevalência obtida nos trabalhos científicos deve ser considerada alarmante e relevante devido à atrocidade que esse tipo de violação representa.

Nos Estados Unidos, o abuso sexual infantil é a terceira causa mais comum de violência contra as crianças, depois da negligência e do abuso físico ^20^ . Dados nacionais do 13° Anuário de Segurança Pública indicam que em 2018 foram registrados no Brasil 66.041 casos de estupro (média de 180 casos/dia); 80,0% das vítimas eram do sexo feminino e 53,8% tinham menos de 13 anos de idade, ou seja, quatro meninas de até 13 anos foram estupradas por hora no ano em questão ^21^ .

Neste estudo, 8,3% dos alunos relataram ter sofrido violência sexual em algum momento da infância ou adolescência, sendo 73,2% meninas e 26,8% meninos. Acredita-se que esses números possam estar subestimados, considerando a recusa de alguns alunos em participarem desta investigação. Congruente com o que foi encontrado, trabalhos mostram que a violência sexual é predominante no sexo feminino ^3^^,^^21^^,^^22^ . Acredita-se que a subnotificação entre a população masculina seja muito expressiva ^23^^,^^24^ . Normas sobre masculinidade podem influenciar a decisão dos meninos quanto a revelar o histórico, por temerem ser tachados como homossexuais ^23^^,^^24^ . Ser vítima de abuso coloca os meninos em posição de grande fragilidade, o que está em completo desacordo com o construto dominante da masculinidade imposto pela sociedade ^23^^,^^24^ .

Foi observado, na população estudada, predomínio das classes socioeconômicas A, B1 e B2, revelando que, apesar de existir a política de cotas para o ingresso em universidades públicas desde 2012, ainda há importante diferença no acesso das classes menos favorecidas. Embora tenham sido encontradas vítimas de violência em todas as classes sociais e de haver neste estudo apenas um indivíduo pertencente à classe D/E, observou-se franco predomínio da violência sexual em camadas com menor poder aquisitivo. Pelos resultados encontrados, pode-se pensar na possibilidade de haver fatores ligados à pobreza que colocariam esses indivíduos em situação de maior risco e menor proteção. Carneiro e Veiga ^25^ dizem que a pobreza expõe os indivíduos a situações de risco, principalmente em contextos nos quais não se pode contar com uma rede de suporte social que ofereça instrumentos para o enfrentamento das adversidades. Não é possível afirmar que o nível socioeconômico seja fator de risco direto para a violência sexual, visto que, de acordo com a literatura, é um tipo de violência que acomete todas as classes sociais de maneira igualitária, mas se pode refletir que famílias com carência de recursos financeiros podem estar mais propensas a deixar suas crianças em situações de risco, negligenciando seus cuidados, muitas vezes por falta de melhores opções. Uma revisão da literatura publicada em 2018 afirma que crianças do sexo feminino em famílias cujas rendas estão abaixo ou no limiar da pobreza correm maior risco de serem vítimas, e que a educação parental contribui para esse risco, que é menor em famílias cujas mães têm mais que 12 anos de escolarização ^26^ . A ausência de vagas em creches, de escolas públicas em período integral e de locais gratuitos que possam receber crianças ou adolescentes no contraturno escolar, triste realidade brasileira, obrigam famílias menos favorecidas, que não apresentam recursos financeiros suficientes para pagar por esses serviços, a deixarem suas crianças sob os cuidados de vizinhos e familiares com poucas condições de supervisionar adequadamente, ou mesmo sozinhos em casa ou sob os cuidados de irmãos pouco mais velhos. Dessa forma, as crianças ficam expostas a situações de risco muitas vezes identificadas pelo perpetrador, que geralmente é alguém do convívio da vítima. O algoz enxerga a falta de segurança que rodeia a vítima.

Interessante notar que os alunos vítimas de violência sexual aqui estudados eram mais velhos do que os não vítimas, ou seja, o ingresso na universidade por essa população ocorreu mais tardiamente. Pode ser que, por serem menos favorecidos economicamente, esses sujeitos sejam provenientes de escolas públicas ou privadas mais populares, cujo ensino não basta para o ingresso em universidades muito concorridas, o que requer anos de aulas preparatórias para conseguir entrar nos cursos em que estavam matriculados. Sabe-se quão caras são as escolas cujo ensino é adequado e prepara os alunos para ingresso em universidades públicas no Brasil. Por outro lado, é descrito que a violência sexual pode gerar grande impacto na vida acadêmica da vítima ^27^ . Alterações no desenvolvimento cognitivo, na linguagem, na memória e no rendimento escolar, além de rebaixamento da percepção do próprio desempenho e na capacidade, têm sido frequentemente relatados ^27^^,^^28^ . Embora todos os sujeitos desta pesquisa sejam universitários, pode-se refletir que o prejuízo escolar advindo do abuso obrigou as vítimas de violência sexual a fazerem mais anos de cursos preparatórios para ingressar na universidade, o que repercutiu na idade de ingresso desses jovens no ensino superior.

Merece destaque a relação entre a ocorrência de violência sexual na infância e adolescência e o comportamento sexual. Encontraram-se no grupo vítima de violência mais alunos que já haviam tido a coitarca e alunas que já haviam engravidado, sugerindo que as meninas vítimas de violência são mais sexualmente ativas quando comparadas às não vítimas, corroborando o que sugere a literatura sobre a vivência de abuso sexual e o desenvolvimento de comportamento sexual de risco, como atividade sexual desprotegida, precoce, múltiplos parceiros e promiscuidade ^29^^,^^30^ . Estudos têm mostrado relação entre abuso sexual na infância em mulheres com sexarca mais precoce, maior quantidade de parceiros, risco aumentado do uso de substâncias psicoativas antes da prática sexual, maior número de relações desprotegidas, além de maior prevalência de IST ^29^^,^^30^ . Há evidências também do aumento de comportamentos sexuais de risco nos indivíduos do sexo masculino que sofreram abuso e têm relações homossexuais, como a prática de relação anal desprotegida ^24^ .

Quando se comparou a presença de sintomas depressivos e ansiosos, qualidade de vida e uso de álcool, tabaco e outras substâncias entre a população vítima de violência com a população que não foi violada, encontraram-se resultados compatíveis com os da literatura. Os sobreviventes de violência sexual apresentaram maiores escores para depressão, ansiedade e pior qualidade de vida quando comparados aos que não foram vítimas desse tipo de violência. Em relação ao uso de drogas lícitas ou ilícitas, observou-se que os indivíduos vítimas de abuso apresentaram maior consumo de tabaco e maconha e usaram de maneira abusiva sedativos ou hipnóticos, talvez para minimizar o desconforto dos sintomas depressivos ou ansiosos. É bem descrito que a violência sexual é grande fator de risco para o desenvolvimento de depressão, ansiedade, TEPT, problemas de sono, fobias, transtornos alimentares, tentativa ou ideação suicida, automutilação e abuso de drogas, com depressão e TEPT permanecendo as formas mais comuns de problemas mentais associados ao abuso sexual ^1^^,^^3^ . Quando a violência sexual acomete crianças e adolescentes, torna-se especialmente dramática, visto que ameaça o desenvolvimento sexual e psíquico de um indivíduo em formação ^4^^,^^6^ . Crianças vítimas de violência sexual estão mais sujeitas ao uso de tabaco, abuso de álcool ou drogas, comportamento sexual de risco (início precoce da atividade sexual consensual, múltiplos parceiros e intercurso sexual desprotegido), timidez, isolamento, vulnerabilidade a se tornarem vítimas novamente desta ou de outras formas de violência, problemas acadêmicos, delinquência, baixa autoestima, agressividade, condutas autodestrutivas, desesperança em relação ao futuro, mentiras, dificuldade em confiar nos outros e danos à qualidade de vida ^1^^–^^3^^,^^6^^,^^9^ .

É relevante considerar que muitas das violações vividas pelos sujeitos da pesquisa podem ter ocorrido na infância ou adolescência, períodos um pouco distantes do momento da realização deste trabalho. Vê-se que as consequências negativas do abuso podem persistir por um longo prazo. Importante frisar que essa população não foi recrutada em serviços de apoio à vitimas de violência, o que poderia ser um viés nos resultados apresentados, visto que os indivíduos normalmente procuram e são acompanhados nesses serviços por necessitarem de suporte e apoio, ou seja, por estarem, de certa forma, mais fragilizados e propensos a estarem deprimidos, com sintomas ansiosos e com sensação de pior qualidade de vida ou predispostos a utilizar mais álcool e outras drogas.

Tendo em vista os resultados aqui obtidos, prevenir que a violência sexual contra a criança e o adolescente ocorra deve ser o objetivo maior de todos os programas de saúde pública. Esse tipo de brutalidade deveria chocar a sociedade e ser tratada como crime hediondo. Apenas dessa forma passaria a não ser tolerada e, no caso de ocorrência, seria prontamente denunciada.

Devido ao ônus que acarreta para os sobreviventes, para os sistemas de saúde e para a sociedade como um todo, deve ser assunto de grande interesse científico e popular. Acredita-se que a educação e a produção de material sobre o tema possam mobilizar, sensibilizar e instrumentalizar, pais, crianças, pares, educadores, profissionais de saúde, enfim, todo o coletivo, desmistificando o assunto e chamando atenção para essa importante questão social.

É pertinente chamar atenção para o fato de que todos os participantes da pesquisa são indivíduos que estão regularmente matriculados em uma universidade pública do Brasil, instituição com concorrência acirrada para ingresso, ou seja, são sujeitos reconhecidamente capazes e inteligentes, que, apesar dos dramas vividos, estão seguindo em frente.

Este trabalho apontou para a necessidade de dar voz aos sujeitos que foram violados, a fim de conhecer suas histórias de vida, suas angústias, seus caminhos, suas superações. Está se dando continuidade a este estudo, utilizando abordagem qualitativa, por obtenção da história oral dos indivíduos que foram vítimas de abuso sexual.
